# Neurocognitive processing of infant stimuli in mothers and non-mothers: psychophysiological, cognitive and neuroimaging evidence

**DOI:** 10.1093/scan/nsab002

**Published:** 2021-01-09

**Authors:** Anne Bjertrup, Nellie Friis, Mette Væver, Kamilla Miskowiak

**Affiliations:** Copenhagen Affective Disorders research Center (CADIC), Psychiatric Centre Copenhagen, Mental Health Services, Capital Region of Denmark, 2100 Copenhagen, Denmark; Department of Psychology, Faculty of Social Sciences, University of Copenhagen, 1355 Copenhagen, Denmark; Copenhagen Affective Disorders research Center (CADIC), Psychiatric Centre Copenhagen, Mental Health Services, Capital Region of Denmark, 2100 Copenhagen, Denmark; Department of Psychology, Faculty of Social Sciences, University of Copenhagen, 1355 Copenhagen, Denmark; Center for Early Intervention and Family Studies, Department of Psychology, Faculty of Social Sciences, University of Copenhagen, 1355 Copenhagen, Denmark; Copenhagen Affective Disorders research Center (CADIC), Psychiatric Centre Copenhagen, Mental Health Services, Capital Region of Denmark, 2100 Copenhagen, Denmark; Department of Psychology, Faculty of Social Sciences, University of Copenhagen, 1355 Copenhagen, Denmark

**Keywords:** fMRI, neural plasticity, caregiving, mother–infant relations, postpartum

## Abstract

Emerging evidence indicates that mothers and non-mothers show different neurocognitive responses to infant stimuli. This study investigated mothers’ psychophysiological, cognitive and neuronal responses to emotional infant stimuli. A total of 35 mothers with 4-month-old infants and 18 control women without young children underwent computerized tests assessing neurocognitive processing of infant stimuli. Their eye gazes and eye fixations, galvanic skin responses (GSRs) and facial expressions towards infant emotional stimuli were recorded during the tasks. Participants underwent functional magnetic resonance imaging during which they viewed pictures of an unknown infant and, for mothers, their own infants. Mothers gazed more and had increased GSR towards infant stimuli and displayed more positive facial expressions to infant laughter, and self-reported more positive ratings of infant vocalizations than control women. At a neural level, mothers showed greater neural response in insula, dorsolateral prefrontal cortex and occipital brain regions within a predefined ‘maternal neural network’ while watching images of their own *vs* unknown infants. This specific neural response to own infants correlated with less negative ratings of own *vs* unknown infants’ signals of distress. Differences between mothers and control women without young children could be interpreted as neurocognitive adaptation to motherhood in the mothers.

## Introduction

Infant faces rapidly attract the attention of parents as well as non-parents (Kringelbach *et al.*, 2016). But research indicates that mothers’ attention is captured by infant stimuli to a greater extent than non-mothers’ attention (Thompson-Booth *et al.*, 2014a,b). Our recent systematic review of research into mothers’ neural responses to infant stimuli showed that mothers display generally ‘faster attention allocation’ to infant stimuli than non-mothers, as indicated by electrophysiological brain responses (Bjertrup *et al.*, 2019). Accumulating longitudinal evidence suggests that the transition to motherhood is associated with neural reorganization (Hoekzema *et al.*, 2017, 2020). This may be supported by neuroendocrine and psychological changes throughout pregnancy and peripartum (Henry and Sherwin, 2012) and through experience with the infant after birth may consolidate the neural architecture that supports adaptive maternal behaviour (Parsons *et al.*, 2017b). These longitudinal studies have reported that changes in structure and function of brain areas associated with social cognition (Hoekzema *et al.*, 2017), reward (Hoekzema *et al.*, 2020) and attention (Dudek *et al.*, 2020) from pregnancy to postpartum predict maternal behaviours (Hoekzema *et al.*, 2017, 2020) and mother–infant bonding (Dudek *et al.*, 2020). Therefore, putative differences between mothers and non-mothers in neurocognitive processing of infant stimuli could—in the absence of differences in demographic variables—reflect neurocognitive changes associated with motherhood consistent with previous research. Alternatively, it may reflect initial differences in motivation to become a mother.

Consistent evidence indicates enhanced neural responses in mothers to their own *vs* unknown infants in several regions, including middle frontal gyri (MFG), orbitofrontal cortex (OFC), insula and precuneus (Bjertrup *et al.*, 2019). These regions are part of a ‘maternal neural network’ supporting healthy maternal functions including attention, emotion, regulation, empathy, motivation and reward processing (Bjertrup *et al.*, 2019).

Sensitive and contingent maternal responses appropriately attuned to the infant’s signals are vital for the infant’s maturing capacity for self-regulation (Fonagy *et al.*, 2007). Whereas infants’ signals of distress ensure that physiological needs are met, infants’ positive signals likely serve to keep in proximity to the caregiver (Bowlby, 1997). Some evidence indicates that intense infant distress captures the attention of adults to a greater extent than other expressions, which emphasizes the evolutionary importance of these stimuli (Lucion *et al.*, 2017). Intense infant distress can be highly stressful for caregivers (Lingle, 2019). In spite of this, how is it that infant signals of distress evoke caregiving responses rather than aversion? One explanation could be that mothers perceive infant characteristics as more rewarding than women without young children do and are therefore less negatively affected by (and more likely to cope with) intense infant distress. Importantly, mothers’ more positive neurocognitive response to infant signals may aid maternal behaviour in real-life interactions with their infants. Indeed, attuned enthusiastic and joyful maternal facial expressions when infants express positive emotions are important for stimulating playful mother–infant interactions and thus the child’s sense of social connectedness (Feldman, 2003). Motherhood may entail differences from non-motherhood in neural, cognitive and behavioural responses to infants at psychophysiological, neural, cognitive and relational levels. However, no previous study has performed an integrated comparison of mothers and non-mothers across these multiple measures.

This study investigated mothers’ psychophysiological and cognitive responses to emotional infant faces and vocalizations and mothers’ neural responses to own *vs* unknown infants’ emotional faces. We hypothesized that compared to control women, mothers would show (i) greater visual attention and physiological reactivity towards emotional infant stimuli and (ii) a positive bias to infant stimuli as reflected by more positive facial expressions to happy *vs* distressed infant faces and vocalizations and more positive and less negative ratings of infant facial and vocal expressions of happiness and distress, respectively. We also hypothesized that mothers would (iii) show greater neural responses to their own *vs* unknown infant faces within regions of the maternal neural network and that this would correlate with more positive and/or less negative ratings of own *vs* unknown infants. Finally, we explored whether mothers and control women would show differential neural responses to distressed *vs* happy infant faces.

## Methods and materials

### Participants

Mothers were recruited from the Department of Obstetrics and Gynecology, Hvidovre Hospital, Denmark, or through print and online advertisements. Control women were recruited through advertisements. General inclusion criteria were age ≥18, no personal history of mental illness, assessed by the Mini International Neuropsychiatric Interview (MINI) (Sheehan *et al.*, 1998), no neurological illness or current alcohol or substance abuse (defined by ICD-10 F10.1 or F10.2 criteria), no indication of personality disorder (defined by a total score of ≤3 on the Standardised Assessment of Personality—Abbreviated Scale (SAPAS) (Hesse and Moran, 2010)), no major psychiatric disorder among first-degree relatives, no magnetic metal implants (MR contra-indications) and, for mothers’ infants, no diagnosis of Down’s syndrome, cerebral palsy or other severe neurological illnesses. An exclusion criterion for control women was children aged <6 years. The project was approved by the local ethics committee in the Capital Region of Denmark (ID: H-17009045) and by the Danish Data Protection Agency Capital Region of Denmark (ID: RHP-2017-024; I-Suite: 05603). Written informed consent was obtained from all participants. The study was conducted in accordance with the Declaration of Helsinki.

### Experimental design

Mothers were assessed 4 months (mean ± SD: 4.0 ± 0.4) after birth during a 4-h home visit and participated in a one-and-a-half-hour functional magnetic resonance imaging (fMRI) scan session at another day separate from the home visit. Control women went through one test session with duration of 4 h at Rigshospitalet, Copenhagen. For all participants, the presence of mild depression symptoms was assessed with the Hamilton Depression Rating Scale-17 items (HDRS-17) (Hamilton, 1967) and self-assessed state and trait anxiety was measured with the State-Trait Anxiety Questionnaire (STAI) (Spielberger, 1983). Non-emotional cognition was assessed with the short (<20 min) Screen for Cognitive Impairment in Psychiatry (SCIP) (Purdon, 2005; Ott *et al.*, 2016) and all infant emotion processing tasks were computerized. Participants’ facial expressions, galvanic skin responses (GSRs), eye gazes and fixations in response to infant emotional stimuli were recorded during the infant emotion processing computer tasks. Participants went through an fMRI scan where they watched an unknown 4-month-old Caucasian infant girl’s face and mothers additionally viewed their own infant’s face (images obtained during the home visit). After the scan, participants were asked to rate the infant faces according to how they thought the infant was feeling and how they themselves felt when watching the infant faces. Mothers and control women went through an fMRI scan, where they completed an ‘adult face emotion processing task’, which will be reported elsewhere. The sample of mothers in the current study was also compared with mothers with affective disorders on emotional cognition and neural activity [results reported elsewhere (Bjertrup *et al*., in review-a,b)].

### Infant emotion processing (I): behavioural and psychophysiological measures

Infant stimuli were presented on a Lenovo T430 14″ laptop with 1920 × 1080 resolution monitor using iMotions Software version 6.4 and integrated hardware to record psychophysiological responses (iMotions A/S, Copenhagen, Denmark).

#### Infant emotion rating task.

Participants were asked to rate the emotional intensity of 50 infant faces and 50 infant vocalizations. For both stimuli types, infant emotion was expressed at five intensities: most happy, moderately happy, neutral, moderately distressed and most distressed. The face images displayed infants aged 3–14 months (Kringelbach *et al.*, 2008) and infant vocalizations consisted of sound recordings from real-life parent–infant interactions (Parsons *et al.*, 2014). Vocalizations were presented either through computer speakers or headphones, while the screen turned black. Infant stimuli were presented for 2 s. Between each stimuli presentation, a horizontal rating bar appeared on the screen and participants had a maximum of 5 s to rate the infant emotion on a continuous Likert scale ranging from −4 (infant most distressed) to +4 (infant most happy).

#### Infant videos.

In order to record ‘natural’ psychophysiological responses to infant emotional stimuli, not interfered by a task and of longer duration than the images and vocalizations mentioned above, participants were instructed to passively watch two infant videos—a ‘laughter’ and ‘distress video’—of 28 s each. The videos were presented once to each participant and in a random counterbalanced order between participants. The ‘laughter video’ showed infant quadruplets and their mother laughing continuously throughout the video. The ‘distress video’ displayed a distressed infant boy crying intensely without being picked up or comforted.

#### Psychophysiological responses to infant stimuli.

Participants’ facial expressions displayed in response to infant emotional stimuli were recorded by the laptop’s webcam and post-processed with Affectiva Affdex in the iMotions software. The Affectiva Affdex algorithm uses the Facial Action Coding System to identify and categorize facial expressions as positive or negative based on specific facial ‘action units’ (Ekman and Friesen, 1978; Affectiva, 2017; iMotions, 2017b). Results were reported as percent of time a participant showed a positive or negative facial expression. The iMotions software estimated the probability that the expression determined by the algorithm was equal to the evaluation of a human rater and discarded probabilities below 10%. Therefore, expressions with high uncertainty were reported as ‘0% of time’ for both positive and negative emotions (Affectiva, 2017; iMotions, 2017b). Eye gaze and eye fixation at the computer screen displaying infant stimuli were tracked with a Tobii Pro 60 Hz eye tracker (Tobii Pro, Sweden) mounted on the test computer. The eye tracker directs near-infrared light towards the participant’s pupils and records eye movements from reflections of the cornea (Gonzalez-Sanchez *et al.*, 2017; iMotions, 2017a). Eye movements with a velocity <30 degrees per second were classified as fixations by a Velocity-Threshold Identification (I-VT) fixation algorithm in the iMotions software (iMotions, 2018), whereas gaze time was defined as total time spent looking at stimuli. Short fixations (<60 ms) were excluded from the data. We investigated time spent gazing and fixating at infant faces by defining these as areas of interest (AOIs) for the analyses. Thus, gazes and fixations outside the AOIs, for example the background or the mother in the ‘laughter video’, were discarded. To enhance measurement accuracy, the eye tracker was calibrated to participants’ eye movements before each task (Tobii Pro, 2020). GSR to infant emotional videos was measured with a Shimmer Sensor3 with 128 Hz sampling rate by two electrodes attached to participants’ index and ring fingers. The sensor was connected via Bluetooth to iMotions software where a ‘peak detection algorithm’ detected GSR peaks defined as signals crossing a threshold of 0.01 μS and with an amplitude of at least 0.005 μS. Participants’ GSRs were defined as number of peaks per minute. Since infant sounds and images were only presented for 2 s, and GSR signals are delayed by 1–5 s (Benedek and Kaernbach, 2010), we only included GSRs to infant videos and only responses occurring later than 1000 ms. For eye tracking, the individual data points of low quality (under 70%) were excluded from analyses.

### Infant emotion processing (II): neural responses to own *vs* unknown and distressed *vs* happy infant faces

#### fMRI paradigm.

Images of mothers’ own infant’s face were obtained during the home visit, and the three pictures with most happy expressions and three with the most distressed expressions were selected for the scan. Images were standardized according to size, orientation and lighting and edited in GNU Image Manipulation Program (GIMP) v. 2.8.22 (GIMP Development Team, 2017) so only faces were visible and placed on a black background. A block consisted of three images belonging to the same category, for mothers: (i) own happy, (ii) own distressed, (iii) unknown happy and (iv) unknown distressed and for control women: (i) unknown happy and (ii) unknown distressed. Images within a block were shown for 3750 ms and separated by a 500 ms fixation cross. The blocks were shown six times in a pseudorandomized order separated by a 2500 ms fixation cross. The paradigm was created and run in E-prime version 2.0 software (Psychology Software Tools) and projected onto an opaque screen placed at the head end of the scanner and from there visible to participants on an overhead angled mirror inside the scanner. Participants were instructed to simply watch the images. After the scan participants rated how they thought the infants were feeling and how they themselves felt when looking at the images on a scale from −4 (most distressed), 0 (neutral) to +4 (most happy) with nine possible answers.

#### fMRI data acquisition.

Neural activation to the infant paradigm was assessed with *in vivo* non-invasive techniques of fMRI using a 3T Siemens MR scanner and a 64-channel head–neck coil. The total duration of the scanner sequence was 45 min and included: localizer, high-resolution T1-weighted structural images of the whole brain [T1 sequence: MPRAGE, echo time (TE) = 2.58, repetition time (TR) = 1900 ms, flip angle 9°, distance factor = 50%, a 230 × 230 mm field of view (FOV) and slice thickness = 0.9 mm], and T2*-weighted gradient echo spiral echo-planar imaging sequence (TE = 30 ms, TR = 2 s and flip angle = 90°). A total of 193 brain volumes, consisting of 32 slices with slice thickness of 3 mm and 25% gaps in between (FOV of 230 × 230 mm using a 64 × 64 grid) were acquired. A standard B0 field map sequence (230 × 230 mm FOV; TR = 400 ms; TE = 7.38 ms; flip angle = 60°) was obtained to enable correction for geometric distortions. Mothers went through an additional functional paradigm (used for another study) and lastly a 10-min resting state scan. Analyses of fMRI data were carried out in the FMRIB Expert Analysis Tool (FSL v. 6.00.) (https://www.fmrib.ox.ac.uk/fsl).

### fMRI data analysis

Data were visually inspected for artefacts and excessive movement. Preprocessing included removal of non-brain tissue with FSL brain extraction tool (Smith, 2002), and realignment, normalization, spatial smoothing using a 5-mm full-width at half-maximum Gaussian kernel and motion correction with MCFLIRT. Motion outlier peaks with mean displacement >1 mm, indicated excessive head movement, and these single volumes were removed with MCFLIRT motion correction. Volumes were registered to MNI152 standard space and registrations were visually inspected. In FSL FEAT, five and three explanatory variables (EVs) were modelled for mothers and control women, respectively, using a general linear model (GLM). The five EV conditions (i) own happy, (ii) own distress, (iii) unknown happy, (iv) unknown distress, (v) intertrial fixation crosses were modelled for mothers, while (i) unknown happy, (ii) unknown distress and (iii) intertrial fixation crosses were modelled for control women and convolved with double-gamma hemodynamic response function and included temporal derivatives. Six contrasts (i) own *vs* unknown, (ii) unknown *vs* own, (iii) own happy, (iv) own distress, (v) unknown happy and (vi) unknown distress were defined in single subjects analyses for mothers. In addition, three contrasts—(i) distressed infants, (ii) happy infants and (iii) distressed *vs* happy infants—were defined in single-subject analyses for mothers and control women. The higher-level analysis of mothers’ blood-oxygen-level dependent (BOLD) activation in response to the contrasts (i) own *vs* unknown and (ii) unknown *vs* own and of all participants’ BOLD response to infant distress *vs* happiness were run in FSL FEAT using a mixed-effects model (FLAME 1).

We investigated activation differences in an a priori defined ‘volume-of-interest’ (VOI), which included ‘maternal neural network’ structures previously identified as responsive to ‘own *vs.* unknown’ emotional infant faces, namely STG, OFC, insula, striatum, fusiform gyrus, precuneus and the dlPFC structure MFG (see Bjertrup *et al.*, 2019 for a review of existing studies). Structures were obtained from Harvard–Oxford cortical and subcortical structural atlases in FSLeyes, thresholding at 25%, binarized and combined in one VOI mask, which was uploaded as pre-threshold masking for small-volume correction in the higher-level FEAT analysis. Amygdala was investigated as a single ‘region-of-interest’ by retrieving left and right amygdala from the Harvard–Oxford subcortical structural atlas in FSLeyes, thresholding at 25% in order to exclude voxels that have 25% or less probability of belonging to that region. Mean percentage BOLD signal change to own and unknown infant happy and distressed faces in the left and right amygdala was extracted by applying the amygdala mask during FEATquery. Bilateral amygdala responses to own and unknown infants were compared with paired samples *t*-test (level of alpha = 0.05). Finally, we conducted an exploratory whole-brain analysis. The significance level for clusters was set to *P* < 0.05 corrected for multiple comparisons using Gaussian Random Field following a cluster-forming threshold of *z* = 2.57 (uncorrected *P = *0.005) for all higher-level analyses conducted in FSL FEAT. Location of peak cluster foci were identified with Harvard–Oxford cortical and subcortical structural atlases in FSLeyes, and Brodmann Areas were identified using the Talairach Atlas (Talairach, 1988).

#### Association between neural response to and rating of infants.

For structures with significantly increased activation to own *vs* unknown infant and to distressed *vs* happy infant faces, we extracted mean percent BOLD signal changes in response to the individual images (own happy, unknown happy, own distress and unknown distress as well as all distressed and all happy) using the FEATquery tool in FSL. The mean percent BOLD signal change to own *vs* unknown infants and distressed *vs* happy infants were correlated with participants’ post-scan ratings of own *vs* unknown infants’ happy and distressed faces, all distressed *vs* happy infant faces and the own emotional response while viewing these images.

### Statistical analyses

Group differences in demographic variables, subsyndromal depression symptoms, personality traits and non-emotional cognition were investigated with *t-*tests. Group differences in psychophysiological measures, infant emotion ratings and state and trait anxiety were analysed with repeated-measures analysis of variance with infant emotion or subscale (trait or state anxiety subscale) on the STAI questionnaire as within-subjects factors and group as between-subjects factor. Significant interaction effects were followed up by *t-*tests for normally distributed data, whereas Mann–Whitney U tests were used for non-normally distributed data. *Post hoc* control for any significant group differences on depression or anxiety scales were performed by including scales as covariates in analyses for psychophysiological measures and infant emotion ratings. Differences in participants’ post-scan ratings of own compared to unknown infant faces, all distressed *vs* happy infant faces and of their own emotional response to infant faces were investigated with paired samples *t-*test. Differences in ratings for own *vs* unknown infants’ happy and distressed faces were analysed separately. The association between signal changes in significant clusters and post-scan ratings were explored with Pearson’s correlation analyses. Due to the exploratory nature of this study, the *P-*values were not corrected for multiple comparisons in the primary analyses. However, for significant differences between groups we conducted *post hoc* adjustment for multiple comparisons with Benjamini–Hochberg (B-H) correction to examine the robustness of the results (Benjamini and Hochberg, 1995). Data were analysed using the Statistical Package for the Social Sciences version 25 (IBM Corp., 2017).

## Results

### Participant characteristics and non-emotional cognition

A total sample of 35 mothers and 18 control women were assessed for this study. As seen from Table 1, mothers and control women were well-matched for age (*P = *0.67), years of education (*P = *0.21), employment status (*P = *0.16), subsyndromal depression symptoms on HDRS-17 (*P = *0.65), subclinical dysfunctional personality traits (SAPAS) (*P = *0.19) and non-emotional cognition (SCIP total score: *P = *0.32). However, mothers were more often living in a relationship (*P* ≤ 0.001) and displayed less trait anxiety than control women (*t = *2.11, df = 51, *P = *0.04). One control woman had two children >6 years. Both primiparous and multiparous mothers were included. On average, the mothers participated in the fMRI session 7 days after the home visit (days, median: 5, IR: 6). While 33 mothers participated in the fMRI scan between 2 and 45 days after the home visit, one mother, went through the fMRI scan 4 days ‘before’ the home visit for practical reasons and therefore provided her own photos of her infant.

**Table 1.** Participant demographics and clinical information

	Mothers	Control women	F/*t*/Chi-square	*P-*value*
	*N* = 35^a^	*N* = 18		
Age, years, mean (s.d.)	30.7 (3.3)	31.2 (4.3)	0.43	0.67
Years of education, median (IR)	17.0 (3.0)	18.0 (2.0)	1.28	0.21
Occupation				
Employed, *n* (%)	31 (88.6)	12 (66.7)	3.70	0.054
Student, *n* (%)	4 (11.4)	5 (27.8)	2.25	0.13
Living with partner, *n* (%)	32 (91.4)	8 (44.4)	14.18	**≤0.001**
Right-handed, *n* (%)	33 (94.3)	18 (100.0)	1.07	0.30
HDRS-17, median (IR)	2.0 (2.0)	1.5 (2.0)	0.46	0.65
SAPAS, median (IR)	1.0 (1.0)	1.0 (2.0)	1.33	0.19
State anxiety, mean (s.d.)	47.0 (2.8)	45.4 (3.9)	1.67	0.10
Trait anxiety, mean (s.d.)	43.9 (3.5)	46.0 (3.1)	2.11	**0.04**
SCIP total, mean (s.d.)	83.0 (6.6)	80.8 (8.2)	1.01	0.32
Breastfeeding, *n* (%)	31 (88.6)	NA	NA	NA
Parity, median (range)	1 (3)	0 (2)	6.98	**≤0.001**
Infant female gender, *n* (%)	15 (42.9)	NA	NA	NA
Infant age, days, mean (s.d.)	120.1 (11.0)	NA	NA	NA
GA, weeks, mean (s.d.)	39.9 (1.4)	NA	NA	NA
Birth weight, gram, mean (s.d.)	3519.7 (723.7)	NA	NA	NA
CS, *n* (%)	5 (14.3)	NA	NA	NA

CS, cesarean section; GA, gestational age; HDRS, Hamilton Depression Rating Scale; NA, not applicable; SAPAS, Standardised Assessment of Personality—Abbreviated Scale; SCIP, Screen for Cognitive Impairment in Psychiatry. Tests for group differences are indicated in the table by Chi-square for categorical data and *t-*tests for numerical data.

a One mother did not wish to go through the MR scan; therefore, the sample size for fMRI analyses is *N* = 34.

* *P-*values for main effect of group. Values display means and standard deviations (s.d.) for normally distributed data and medians and range or interquartile range (IR) or number and percent for non-normally distributed data.

### Infant emotion processing (I): behavioural and psychophysiological measures

#### Assessment of hypothesis 1: maternal vigilance towards infants.

**Fig. 1.**
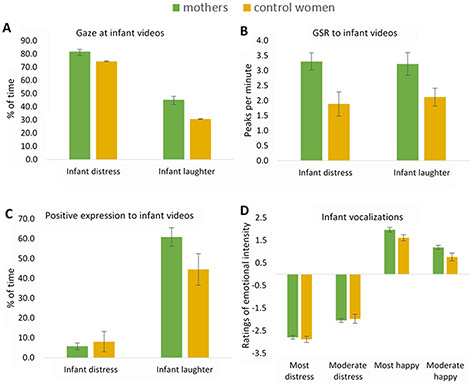
Comparisons of mothers and control women. (A) Percent of time spent gazing at a video of (i) a distressed infant boy crying intensely without being comforted and (ii) infant quadruplets and their mother laughing continuously. Mothers overall spent more time gazing at both the infant distress and infant laughter videos than control women. (B) GSR peaks per minute to the infant distress and infant laughter videos. Mothers had overall more GSR peaks to both infant distress and infant laughter videos than control women. (C) Percent of time displaying positive facial expressions in response to (i) a distressed infant boy crying intensely without being comforted and (ii) infant quadruplets and their mother laughing continuously. An interaction effect indicated that mothers spent a greater percent of time displaying a positive facial expression in response to the infant laughter *vs* distress video than control women. (D) Ratings of infant emotional vocalizations. Mothers generally rated infant vocalizations less negative than control women. Bars show mean scores; error bars show the standard error (standard error of the mean).

Consistent with the hypothesis, mothers spent more time gazing at infant videos [mean: mothers: 64.4%, control women: 52.3%; F(1,44) = 19.35, *P* ≤ 0.001, η^2^=0.31, B-H adjusted *P = *0.01] (Figure 1A) and infant face images than control women [median: mothers: 91.2%, control women: 86.1%; F(1,50) = 6.75, *P = *0.01, η^2^=0.12, B-H adjusted *P = *0.02]. In contrast, they did not spend more time fixating on infant videos or face images (*P-*values ≥ 0.11). Mothers displayed more physiological reactivity to infant videos reflected by more GSR peaks [mean: mothers: 3.2 peaks per minute, control women: 2.1 peaks per minute; F(1,47) = 7.90, *P = *0.01, η^2^=0.14, B-H adjusted *P = *0.02] (Figure 1B). After controlling for trait anxiety symptoms, the main effect of group on gaze time at videos and images and GSR to videos remained significant (video: gaze: *P* < 0.001, GSR: *P = *0.01; face images: gaze: *P = *0.01).

#### Assessment of hypothesis 2: maternal positive bias.

Mothers displayed more positive facial expressions towards the videos of infant laughter *vs* infant cry than control women [interaction effect: F(1, 47) = 4.98, *P = *0.03, η^2^=0.10, B-H adjusted *P = *0.15]. However, *post hoc* Mann–Whitney test showed that this difference in positive facial expression was only a statistical trend [median percent of time displaying positive facial expression, mothers: 0.61%, control women: 0.51%; U = 187.5, *P = *0.08] (Figure 1C). This difference prevailed after *post hoc* adjustment for trait anxiety symptoms (*P = *0.03). Mothers generally rated infant vocalizations as more positive than control women [mean: mothers: −0.42, control women: −0.62; F(1,50) = 4.43, *P = *0.04, η^2^=0.08, B-H adjusted *P = *0.10] (Figure 1D). This was reduced to a statistical trend after adjustment for trait anxiety (*P = *0.059). In contrast, there were no differences in ratings of emotional infant face images (*P-*values ≥ 0.40). There were also no differences between mothers’ and control women’s own emotional expressions to infant face images or vocalizations (*P-*values ≥ 0.18) or negative expressions in response to infant videos (*P-*values ≥ 0.10).

### Infant emotion processing (II): neural responses to infant faces

#### Assessment of hypothesis 3: own infant faces.

**Fig. 2.**
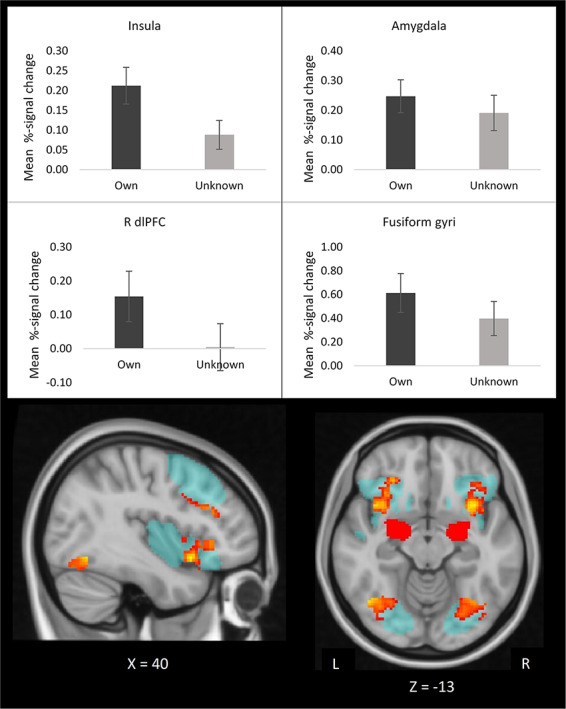
Mothers show enhanced responses in bilateral insula, fusiform gyrus, amygdala and R dlPFC to their own *vs* unknown infants’ faces. The bars display mean percent signal change in response to own and unknown infants. Error bars display standard error of the mean. The brain images display that the significant clusters (yellow-red) lie within the maternal neural network VOI (blue). dlPFC, dorsolateral prefrontal cortex; R, right.

**Fig. 3.**
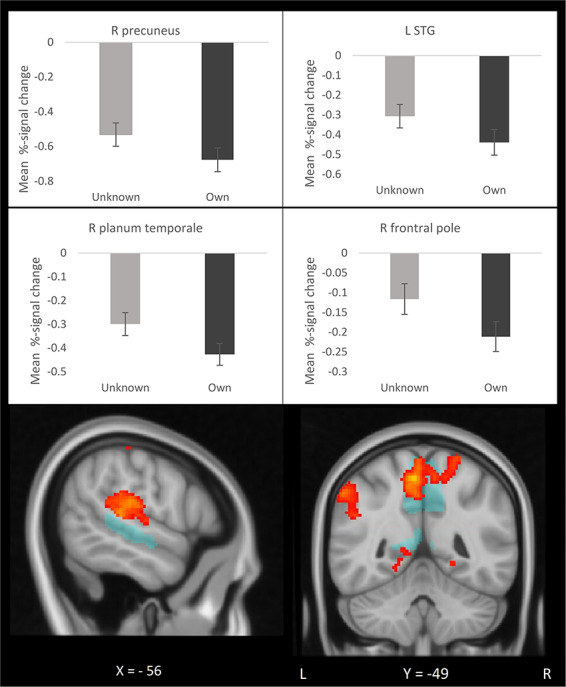
Mothers show ‘more’ deactivations in R precuneus, L STG, R planum temporale and R frontal pole in response to own *vs* unknown infants’ faces. The bars display mean percent signal change in response to unknown and own infants. Error bars display standard error of the mean. The brain images display significant clusters (yellow-red) and their location relative to the maternal neural network (blue). L, left; R, right; STG, superior temporal gyrus.

As hypothesized, mothers showed significantly greater brain activation to their own *vs* unknown infant faces in bilateral insula, fusiform gyrus and right dlPFC, regions of the predefined maternal neural network VOI (Figure 2). Mothers showed more ‘deactivation’ in right precuneus, right frontal pole, right planum temporale and left STG in response to their own *vs* unknown infant faces (Figure 3). Mothers activated the amygdala more in response to own *vs* unknown infant faces (*t = *2.17, df = 33, *P = *0.04) (Figure 2). See Table 2 for fMRI result details.

**Table 2.** Peak cluster activation in VOI and whole-brain regions for mothers in response to watching own and unknown emotional infant faces

Condition	Region	R/L	Peak *Z*	*X*	*Y*	*Z*	BA	*P*	Cluster size (voxels)
*VOI*									
Own > other	Insula	R	5.1	40	14	−12	47	2.7E-09	1300
	Insula	L	5.04	−34	14	−14	47	0.00000453	695
	Occipital fusiform	L	4.72	−26	−68	−18	19	0.00116	334
	Occipital fusiform	R	4.91	44	−62	−16	19/37	0.00208	301
	MFG	R	4.27	44	22	24	9/45/46	0.0184	189
Other > own	Precuneus	R	5.31	4	−56	50	7	5.96E-08	1045
	Frontal pole	R	4.37	36	36	38	8	0.00000143	782
	Planum temporale	R	4.8	66	−18	10	42	0.00324	277
	STG	L	3.74	−68	−36	12	22	0.0192	187
*Whole-brain*									
Own > other	Brainstem	R/L	5.69	0	−18	−14		0	23 651
	Lateral occipital, superior division	L	4.1	−32	−84	18	19	0.00000429	864
	ACC	R/L	3.78	0	38	10	24	0.000297	555
	Precentral gyrus	L	4.65	−42	−12	38	4	0.000445	528
	Precentral gyrus	L	4.82	−46	2	28	6	0.0201	296
	Frontal pole	L	4.46	−8	68	−2	10	0.042	256
	Occipital pole	R	5.44	12	−92	18	18	2.47E-14	2714
Other > own	Precuneus	R	5.31	4	−56	50	7	6.26E-11	1868
	SFG	R	4.54	22	26	60	6	1.02E-10	1819
	Parietal operculum cortex	L	4.36	−58	−26	18	40	1.26E-09	1573
	Parietal operculum cortex	L	4.93	46	−24	16	13	1.62E-09	1549
	Frontal pole	R	5.13	32	60	4	10	0.00000167	940
	Angular gyrus	L	4.14	54	−56	44	40	0.000009	807
	Lingual gyrus	L	4.29	10	−68	0	18	0.000104	627
	Precentral gyrus	L	4.29	−26	−14	68	6	0.00339	399

Coordinates (*x, y, z*) based on Montreal Neurological Institute template refer to the localization of peak activation within a cluster for significant differences in mothers’ neural activation to own compared to unknown infant faces. Significant clusters (corrected *P* < 0.05) are presented with cluster size (voxels) and *Z* statistics for the peak voxel. Regions were identified with Harvard–Oxford cortical and subcortical atlases in FSLeyes, and BAs were identified with Talairach atlas. BA, Brodmann area; L, left; R, right; VOI, volume of interest.

Exploratory whole-brain analyses revealed that mothers displayed greater neural response to own *vs* unknown infant faces in areas across the brain stem, left lateral superior occipital cortex, bilateral anterior cingulate cortex (ACC), left precentral gyrus and left frontal pole—regions that were partially overlapping with the maternal neural network (Table 2). Mothers showed more ‘deactivation’ in medial parts of parietal, occipital and frontal regions in response to their own *vs* unknown infant faces. See Table 2 for fMRI result details.

#### Neural responses to distressed vs happy infant faces in mothers vs control women.

In the maternal network VOI, mothers showed significantly greater response in right dlPFC to (own and unknown) distressed *vs* happy infant faces than control women. Further, the whole-brain analysis showed greater neural response in mothers than control women to distressed *vs* happy infant faces in left middle temporal gyrus (MTG) and right supramarginal gyrus. See Supplementary Table S1 and Supplementary Figure S1 for fMRI result details.

### Ratings of own and unknown infant faces and association with neural responses

Mothers rated their own infants’ distressed faces ‘less negatively’ than unknown infants’ distressed faces (*t = *3.32, df = 33, *P = *0.002), whereas there was no difference in ratings of own *vs* unknown infants’ happy faces (*P = *0.27). Mothers rated their own emotional response to viewing own infants’ happy faces as ‘more positive’ than their feelings in response to unknown infants’ happy faces (*t = *5.09, df = 33, *P* < 0.001). There was a trend towards less negative ratings of own emotional response while viewing own *vs* other infants’ distressed faces (*P = *0.06). The less negative ratings of own *vs* unknown infants’ distressed faces correlated moderately with greater neural activation to own *vs* unknown infant faces in the identified left and right fusiform gyrus (*r* = 0.38, *P = *0.03 and *r* = 0.36, *P = *0.04, respectively) and bilateral ACC (*r* = 3.90, *P = *0.03) and with greater ‘deactivation’ to own *vs* unknown infant faces in right precuneus (*r* = 0.40, *P = *0.02), right frontal pole (*r* = 0.40, *P = *0.02), left lingual gyrus (*r* = 0.38, *P = *0.03), right frontal pole (*r* = 0.49, *P = *0.004), right SFG (*r* = 0.46, *P = *0.01) and right precuneus (*r* = 0.39, *P = *0.03). In contrast, mothers’ positive bias in their own emotional response to viewing infant face images did not correlate with neural activation to own *vs* unknown infant faces (*P-*values > 0.08). There were no differences between mothers’ and control women’s ratings of unknown infant faces (*P-*values > 0.08), and the group differences in neural activation to distressed *vs* happy infant faces did not correlate with ratings of infant faces (*P-*values > 0.07).

## Discussion

This study investigated cognitive, psychophysiological and neuronal responses to infant stimuli in mothers compared with control women without young infants. Consistent with hypothesis (1), mothers gazed more at infant videos and images and display more physiological reactivity while viewing emotional infant videos than control women; a difference that prevailed after *post hoc* B-H correction for multiple comparisons and adjustment for differences between groups in trait anxiety. In support for hypothesis (2), mothers showed a positive bias in their automatic and spontaneous behavioural responses to infants’ emotional signals, as evidenced by more positive facial expressions while viewing an infant laughter video. Mothers also displayed positive bias in their evaluation of infant emotion, as evidenced by positive ratings of infant vocalizations. These findings prevailed after *post hoc* adjustment for trait anxiety but rendered non-significant after *post hoc* B-H correction for multiple comparisona. Finally, consistent with hypothesis (3), mothers displayed greater neural response to their own *vs* unknown infant faces in regions within a broad maternal neural network, including bilateral insula, fusiform gyrus and right dlPFC, and more deactivation of medial frontal, occipital and temporo-parietal regions specifically to own infant images. These neuronal differences correlated with a positive bias in mother’s ratings of own infants’ emotions. Importantly, mothers’ different neurocognitive processing of emotional infant stimuli occurred in the absence of changes in non-emotional cognition.

The findings of increased physiological reactivity and visual attention towards infant stimuli in mothers *vs* control women are consistent with evidence from behavioural (Thompson-Booth *et al.*, 2014a,b) and electrophysiological studies indicating increased and faster attention allocation to emotional infant faces in mothers (Proverbio *et al.*, 2006; Hernandez-Gonzalez *et al.*, 2016). Enhanced attentional processing of infant faces from pregnancy to postpartum has predicted greater mother–infant bonding at 3–5 months postpartum (Dudek *et al.*, 2020). Further, mothers’ elevated ‘skin conductance level’ (i.e. GSR) has been associated with greater maternal sensitivity but only for mothers who also displayed elevated parasympathetic regulation as evidenced by respiratory sinus arrhythmia (Leerkes *et al.*, 2016; Augustine and Leerkes, 2019). Mothers’ increased attention and physiological reactivity may therefore indicate a fast, spontaneous, automatic and adaptive preparedness to instigate caregiving behaviour directed at the needs of the infant.

Mothers’ more positive ratings of infant laughter and less negative ratings of infant cry are consistent with previous observations of positively biased ratings of neutral infant faces in parents *vs* non-parents (Parsons *et al.*, 2017a). While positively biased ratings of infant laughter may indicate increased pleasure, reward and attunement to the infants’ happy emotional state, their lesser negative ratings of infant cries could reflect more capacity to tolerate these highly stressful vocalizations. Finally, the positive bias displayed by mothers in this study overall is consistent with a previous study where parents rated infant cry videos less negatively than non-parents did (Irwin, 2003). The more positive facial expressions in mothers than control women to the infant laughter video could reflect more experiences of pleasure and reward as well as greater emotional attunement, since positive facial expression is congruent to laughter. Such maternal ability to attune facial expressions to the infant in coordinated face-to-face interactions has been shown to support mutual interaction synchrony (Feldman, 2007). This mutual interaction synchrony is a framework for co-regulation of emotional states that promotes infant’s emotional self-regulation and supports emotional and cognitive development (Feldman, 2007). Mother’s less negative ratings of their own *vs* unknown infant’s distressed expressions and more positive ratings of own emotional response to watching own *vs* unknown infant happiness indicate that mothers’ positive bias was most pronounced for their ‘own’ infants specifically. It further signifies an increased ability for tolerating own than unknown infants’ distress and greater experience of pleasure when watching own *vs* unknown infant happiness.

Mothers showed increased amygdala response to own *vs* unknown infant faces, which was also observed in two previous studies (Strathearn *et al.*, 2008; Strathearn and Kim, 2013) and underscores the high personal relevance of own infant faces. Mothers’ increased fusiform gyrus processing of own infant likely reflect familiarity of own infant (Gobbini and Haxby, 2006), in line with previous findings of increased fusiform gyrus response to own *vs* unknown infant faces (Strathearn *et al.*, 2008) and cries (Laurent and Ablow, 2012). Previous studies have also shown increased processing of own *vs* unknown infants in bilateral insula (Strathearn *et al.*, 2008; Lenzi *et al.*, 2009), and given insula’s key role in empathic processing (Carr *et al.*, 2003), mothers may *feel* and reflect on their own infants’ emotions to a greater extent than the unknown infants’ emotions. In keeping with this, Lenzi *et al.* (2009) found that mothers’ increased insula activation to infant emotional faces correlated with greater ability to ascribe mental states to infants. The present study found increased right dlPFC activation in mothers to their own *vs* unknown infant faces, which is consistent with previous findings of enhanced dlPFC processing of own infant emotions (Strathearn and Kim, 2013; Wan *et al.*, 2014) and could indicate top-down regulation of own reactions to infant emotion. This interpretation is in accordance with the interpretation that positively biased ratings of infant distress in these mothers reflect increased ability to tolerate infant distress. That is, increased regulation of own emotional reactions to infant emotions is in line with an increased ability to tolerate infant distress. However, we found no significant correlations between dlPFC response and ratings of infant emotions.

Mothers also displayed greater ‘deactivation’ to own *vs* unknown infants in medial frontal, occipital and temporo-parietal regions (Figure 3)—regions which are largely overlapping with areas within the default mode network (DMN) (Mars *et al.*, 2012; Raichle, 2015). The DMN is active during rest and habitual internal, self-reflective processes and deactivates during externally focused cognitive and emotional processing (Raichle, 2015). Therefore, greater deactivations to own *vs* unknown infant faces in these proposed DMN clusters could indicate greater externally focused attentional processing of own infants specifically. Interestingly, greater activation in medial parietal, occipital and frontal regions of the maternal network correlated with the less negative ratings of their own infants’ distressed faces. Thus, greater attention allocation towards own infants were associated with the maternal positive bias towards their own infants in particular. Greater response of the right dlPFC to distressed *vs* happy infant faces in mothers than control women could indicate enhanced top-down regulation of emotional reactivity to infant distress. Further, the finding that mothers displayed greater neural response than control women in left MTG and right supramarginal gyrus to distressed *vs* happy infant face may be interpreted as greater visual attention towards infant distress in the mothers.

A strength of the study was the integration of psychophysiological, cognitive and neural measures in the investigation of the adaptations to motherhood. Limitations of the study include relatively small sample (*n* = 53) and inequality of the two groups’ sizes (with 35 mothers and 18 control women). Second, the analyses were not adjusted for multiple comparisons and differences in positive attunement to the laughter video and positive ratings of infant vocalizations rendered non-significant after *post hoc* B-H correction. Third, control women reported greater trait anxiety than mothers. However, after adjustment for trait anxiety, the observed differences in facial expression and GSR to videos and gaze time on videos and images prevailed, while only ratings of vocalizations were reduced to a trend. Fourth, we did not investigate whether increased attention towards unknown infants on computer tests was associated with real-life maternal behaviour. Fifth, given the cross-sectional design, we cannot determine whether the observed differences between mothers and control women reflect neurocognitive changes from before pregnancy to motherhood or differences in motivation for motherhood in the two groups. We interpret the observed differences between mothers and control women as neurocognitive changes associated with motherhood based on previous research and the absence of differences between these groups in demographic variables. Yet, given the cross-sectional study design, it is possible that the observed group differences were present before pregnancy and thus represent intrinsic differences in these women’s neurocognitive responses to infants rather than effects of motherhood. Longitudinal assessments of women over a time period and comparisons of neurocognitive changes between women who become mothers and those who do not are warranted to be optimally suited to answer this question. Finally, the fMRI paradigm was suboptimal to test differences between mothers and non-mothers in neural responses to infant faces since mothers saw pictures of both their own and unknown infants, and the fMRI findings should be considered exploratory. However, given the dearth of research in this field, our findings may be hypothesis generating for future research.

In conclusion, the present findings indicate that mothers show heightened vigilance towards infants, faces, more attuned facial expressions and a positive bias in the ratings of infant emotions, especially their own infants’ emotions. Motherhood further involves greater neural processing of own infants’ faces, specifically in regions comprising the functional ‘maternal neural network’ but also in other brain regions, especially those involved in attentional processing. The greater neurocognitive processing of infant stimuli overall seen in mothers *vs* control women may reflect maternal motivation, sensitive caregiving and greater ability in mothers to regulate own emotions and to tolerate infant distress, which together support healthy socio-emotional development in their infants. However, it is plausible that the differences could also reflect differences in motivation to become a mother between these groups. The perspective is insight into healthy neurocognitive adaptations to motherhood, which provides a basis for investigation of potential impairments in mothers with mental disorders. Identification of such impairments has implications for targeted intervention strategies and long-term prevention of intergenerational transmission of risk from these mothers to their children.

## Supplementary Material

nsab002_Supp
