# Supplementary material for: Neurocognitive processing of infant stimuli in mothers and non-mothers: psychophysiological, cognitive and neuroimaging evidence
Source: Soc Cogn Affect Neurosci. 2021 Jan 9;16(4):428–38. doi: 10.1093/scan/nsab002 (PMC7990066; doi:10.1093/scan/nsab002)
Supplement: nsab002_Supp [file nsab002_supp.zip › Supplementary table.docx]

**Supplementary Table 1**

Peak cluster activation in VOI and whole-brain regions for mothers vs. control women in response to watching distressed vs. happy infant faces.

| Condition | Region | R/L | Peak Z | X | Y | Z | BA | P | Cluster size (voxels) |
| --- | --- | --- | --- | --- | --- | --- | --- | --- | --- |
| *VOI* |  |  |  |  |  |  |  |  |  |
| Distress>happy | dlPFC | R | 3.59 | 36 | 30 | 50 | 8 | 0.0411 | 144 |
| *Whole-brain* |  |  |  |  |  |  |  |  |  |
| Distress>happy | Supramarginal gyrus | R | 3.96 | 50 | -44 | 42 | 40 | 0.00179 | 410 |
|  | MTG | L | 4.02 | -54 | -40 | -12 | 20 | 0.0143 | 295 |

Coordinates (x, y, z) based on Montreal Neurological Institute template refer to the localization of peak activation within a cluster for significant differences in mothers’ vs. control women’s neural activation to distressed compared to happy infant faces. Significant clusters (corrected p<0.05) are presented with cluster size (voxels) and  Z statistics for the peak voxel. Regions were identified with Harvard-Oxford cortical and subcortical atlases in FSLeyes and BA were identified with Talairach atlas. Abbreviations: VOI, volumes of interest; BA, Brodmann Area; R, right; L, left; dlPFC, dorsolateral prefrontal cortex; MTG, middle temporal gyrus.
