# Supplementary material for: Neurocognitive processing of infant stimuli in mothers and non-mothers: psychophysiological, cognitive and neuroimaging evidence
Source: Soc Cogn Affect Neurosci. 2021 Jan 9;16(4):428–38. doi: 10.1093/scan/nsab002 (PMC7990066; doi:10.1093/scan/nsab002)
Supplement: nsab002_Supp [file nsab002_supp.zip › Supplementary Figure.docx]

**Supplementary Figure 1**

**Figure.** Comparison of mothers’ and control women’s neural responses to distressed vs happy infant faces. Mothers show enhanced responses in right dlPFC, left MTG and right supramarginal gyrus to distressed vs happy infant faces. Abbreviations: L, left; R, right; MTG, middle temporal gyrus; dlPFC, dorsolateral prefrontal cortex.
